# Molecular profiling of a bladder cancer with very high tumour mutational burden

**DOI:** 10.1038/s41420-024-01883-x

**Published:** 2024-04-30

**Authors:** Manuel Scimeca, Julia Bischof, Rita Bonfiglio, Elisabetta Nale, Valerio Iacovelli, Marco Carilli, Matteo Vittori, Massimiliano Agostini, Valentina Rovella, Francesca Servadei, Erica Giacobbi, Eleonora Candi, Yufang Shi, Gerry Melino, Alessandro Mauriello, Pierluigi Bove

**Affiliations:** 1 Department of Experimental Medicine, TOR, University of Rome “Tor Vergata”, 00133 Rome, Italy; 2 Indivumed GmbH, Falkenried, 88 Building D, 20251 Hamburg, Germany; 3 Urology Unit San Carlo di Nancy Hospital, GVM Care, 00100 Rome, Italy; 4 Department of System Medicine, University of Rome “Tor Vergata”, 00133 Rome, Italy; 5 The Third Affiliated Hospital of Soochow University, Institutes for Translational Medicine, Soochow University, Suzhou, 215000 China

**Keywords:** Predictive markers, Bladder cancer

## Abstract

The increasing incidence of urothelial bladder cancer is a notable global concern, as evidenced by the epidemiological data in terms of frequency, distribution, as well as mortality rates. Although numerous molecular alterations have been linked to the occurrence and progression of bladder cancer, currently there is a limited knowledge on the molecular signature able of accurately predicting clinical outcomes. In this report, we present a case of a pT3b high-grade infiltrating urothelial carcinoma with areas of squamous differentiation characterized by very high tumor mutational burden (TMB), with up-regulations of immune checkpoints. The high TMB, along with elevated expressions of PD-L1, PD-L2, and PD1, underscores the rationale for developing a personalized immunotherapy focused on the use of immune-checkpoint inhibitors. Additionally, molecular analysis revealed somatic mutations in several other cancer-related genes, including TP53, TP63 and NOTCH3. Mutations of TP53 and TP63 genes provide mechanistic insights on the molecular mechanisms underlying disease development and progression. Notably, the above-mentioned mutations and the elevated hypoxia score make the targeting of p53 and/or hypoxia related pathways a plausible personalized medicine option for this bladder cancer, particularly in combination with immunotherapy. Our data suggest a requirement for molecular profiling in bladder cancer to possibly select appropriate immune-checkpoint therapy.

## Introduction

The incidence of urothelial bladder cancer represents a significant global concern, with ever alarming epidemiological data, highlighting its relevance in terms of frequency, distribution, and mortality rates. According to the current statistics, there were over 550,000 new cases and 220,000 deaths of bladder cancer worldwide in 2020, with a substantial impact on public health [1]. Epidemiological analysis reveals significant variations in incidence rates geographically with higher incidence in Europe and North America [2], emphasizing the importance of a detailed assessment of urothelial bladder carcinoma distribution to adopt targeted preventive and therapeutic strategies. From the pathology perspective, urothelial bladder cancer present the most frequent histological type; more rare are the other type such as squamous cell carcinoma, adenocarcinoma and neuroendocrine neoplasms [3, 4].

Recently, the integration of molecular analysis has significantly enriched our understanding of bladder cancer, enabling the identification of predictive and prognostic biomarkers. Mutations in the TP53 gene [5–7] and amplifications of the ERBB2 gene [8–10] have been shown to influence treatment response and prognosis. For instance, patients with specific mutations may have greater benefit from targeted therapies, paving the way for a more personalized approach to bladder cancer management. These molecular-based evidence facilitated the identification of distinct subgroups, each with varying prognostic implications [3, 11, 12]. Molecular subgroups, based on gene expression, exhibit enrichment for specific immunohistochemical phenotypes, distinct genetic profile, as well as immunological patterns. Luminal-papillary subtype shows a higher frequency of FGFR3 mutations, while luminal-unstable basal-squamous, or neuroendocrine-like subtypes are more prone to show TP53 mutations. From a therapeutic standpoint, deregulation of DNA damage repair genes or ERCC2 seems to be correlated with a more positive outcome when treated with cisplatin [13]. Additionally, mutations, amplifications, and fusions involving FGFR3 may be associated with responsiveness to therapy targeting FGFR [14, 15]. The in-depth analysis of molecular features in the context of urothelial bladder cancer reveals additional crucial details for more accurate and personalized disease management. Among the relevant molecular parameters, Tumor Mutational Burden (TMB) [16], chromosomal instability [17], and the expression of immune checkpoints play a central role in patient stratification and in defining therapeutic options. TMB, measured as the total number of genetic mutations in the genome of a tumor cell, has emerged as a predictive indicator of response to immunotherapies. Patients with high TMB are more likely to benefit from therapies based on immune checkpoint inhibition, such as anti-PD-1/PD-L1 antibodies [18]. In the context of bladder cancer, TMB evaluation can provide valuable insights into the potential success of specific immunotherapies [16]. Furthermore, the analysis of immune checkpoint expression, particularly PD-1 and PD-L1, adds crucial information about the immune status of the tumor and its ability to evade the immune system’s response. Bladder tumors expressing high levels of PD-L1 can be ideal targets for immunotherapy, with the potential to enhance the immune response against tumor cells. The integration of TMB, chromosomal instability, and immune checkpoint expression in the molecular analysis of bladder cancer significantly enhances our understanding of the disease. These molecular parameters not only offer more precise diagnostic information but also guide therapeutic choices, opening new perspectives in the personalization of urinary bladder cancer management.

However, at the current state of the art, there is no molecular signature capable of accurately predicting the clinical outcome of urothelial bladder cancers.

In this case report, we describe a case of urothelial bladder cancer with uncommon genomic characteristics. These include a very high TMB, numerous somatic mutations in cancer-related genes, and up-regulation of immune checkpoints, demonstrating the importance of a multi-omics analysis in bladder cancer patients.

## Case presentation and discussion

An 83-year-old female patient with a urothelial neoformation of 80 × 75 × 50 mm allowed histological investigations which classified the lesions as high-grade infiltrating urothelial carcinoma with areas of squamous differentiation, as demonstrated by p40 positivity in immunohistochemistry. The neoplasia infiltrated the wall throughout its thickness, as well as the peri-vesical adipose tissue. Extensively ulcerated areas with associated necrosis were observed. No metastatic lymph nodes were detected. According to the TNM classification, the tumor was staged as pT3b. In addition to p40, immunohistochemical analysis showed a significant positivity for GATA3 or p63. The absence of p16 staining ruled out the involvement of HPV infection in the carcinogenesis.

**Fig. 1:**
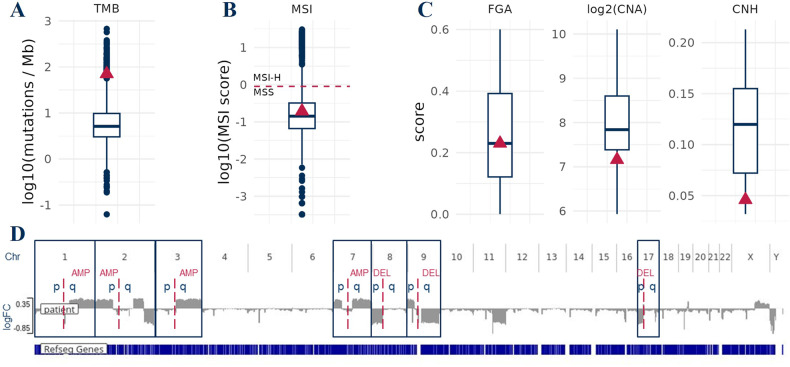
Mutational analysis of an infiltrating urothelial bladder cancer. **A** The patient is high TMB as compared to the average cohort (*n* = 66) of urothelial bladder cancer patients (TMB patient: 70.6). **B** The bladder cancer does not exhibit microsatellite instability (MSS). **C** Graphs display stable chromosome with very low chromosomal copy number heterogeneity. **D** Large chromosomal amplifications in 1q, 2p, 3q, 7q and deletions in 8p, 9q and 17p arm.

Multi-omics investigations revealed a very high TMB (Fig. 1A), no microsatellite instability (Fig. 1B) and low chromosomal copy number heterogeneity (Fig. 1C). Large chromosomal amplifications (>90% of the chromosome arm) in 1q, 2p, 3q, 7q and deletions in 8p, 9q and 17p (Fig. 1D), recently associated to the metastatic potential of uroepithelial tumors [19], have been identified. As compared to the control cohort, the TMB value showed more than a 5-fold increase over the average value (Fig. 1A). TMB has currently considered as a reliable biomarker capable of predicting the response to immune checkpoint inhibitor therapies, mainly anti PD-L1 approaches, in several solid tumors [20–24].

**Fig. 2:**
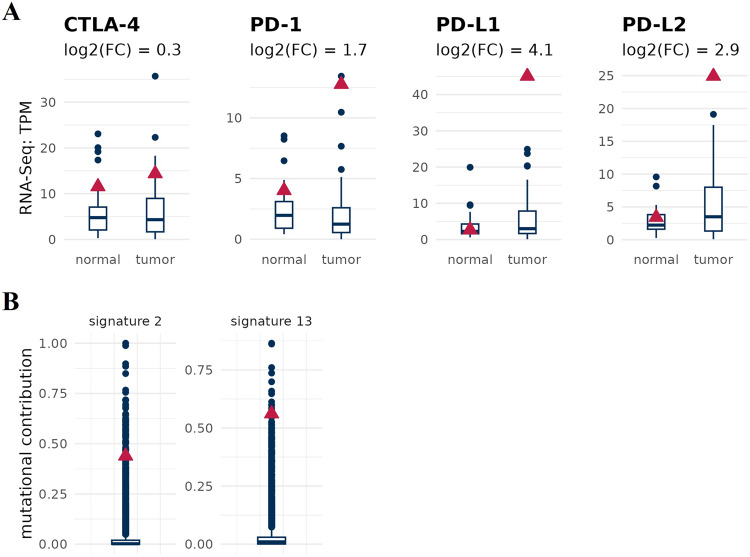
Expression levels of immune checkpoint and mutational signatures in a bladder cancer patient. **A** mRNA expression of PD-1 = Programmed cell death protein-1; PD-L1 and PD-L2 = Programmed death-ligand 1 and 2; CTLA-4 = Cytotoxic T-lymphocyte antigen 4. PD-L1, PD-L2 and PD1 are highly expressed in tumor tissue compared to normal tissue. Boxplots indicate the values of bladder cancer cohort, and the red triangle refers to our patient of interest. **B** Molecular signatures based on global somatic mutation patterns. Analyzed bladder cancer shows high frequencies of mutational signatures Ref Sig 2 + 13. Boxplots indicate the values of bladder cancer background cohort, and the red triangle refers to our patient of interest.

The gene expression analysis also reveals up-regulations of the immune checkpoints PD-L1, PD-L2 and PD1 (PD-1: log2FC = 1.67, PD-L1: log2FC = 4 and PD-L2: log2FC = 2.86) in the analyzed bladder lesion as compared to both normal counterpart and the background cohort of 66 cancer lesions (Fig. 2A). No deregulation of expression was observed for CTLA-4 (Fig. 2A). The concurrent high values of TMB, along with elevated expressions of PD-L1, PD-L2, and PD1, offer clinical evidence for supporting the development of tailored therapies based on immune checkpoint inhibitors [25]. Currently, immunotherapy is a suitable initial treatment for individuals with advanced bladder carcinoma who cannot be treated with chemotherapy using platinum, independently of their immune-checkpoints expression profile [26]. Additionally, for cisplatin-ineligible patientsbut exhibiting high immune-checkpoints expression, immunotherapy could be contemplated as a first-line strategy [27]. More commonly, immunotherapies in bladder cancer are contemplated as second-line strategy for cases with no response, resistance to platinum and were not treated before with immunotherapy [26]. In this scenario, the use of individualized genetic profiles of tumors, as highlighted in this report, will persist in guiding the development of more tailored treatments that are not only better tolerated but also potentially more cost-effective when compared to classical chemotherapy. Whilst the selection of specific immune checkpoint inhibitors remains a topic of debate, significant advantages can be gained from a more detailed molecular profiling analysis of the tumor. As new agents, including the use of antibody-drug conjugates like, continue to be developed and tested, offering plausible perspective to increase bladder cancer treatment options [28–30].

Mutational analysis of our case revealed distinct somatic variations in several cancer related genes (Table 1) such as TP53, TP63, NOTCH3, CDKN2A, CDKN2B, MTAP (see Supplementary Fig. 1).

**Table 1:** Main somatic variation in cancer related genes.

*GENE*	DETAILS
CDKN2A	Delation
*CDKN2B*	Delation
*MTAP*	Delation
*NOTCH3*	Asp 2094 Asn (VAF: 29%)
*TP53*	Lys 139 Asn (VAF: 35.9%)
*TP63*	Arg 141 his (VAF: 24%)

The mutations reported herein in both TP53 and TP63 genes can provide new insights into the underlying mechanisms involved in the development and progression of the disease. TP53, a major onco-suppressor transcription factor, with its family members, is able to directly regulate cell cycle progression [31, 32], metabolic changes [33, 34] and cell death [7] thus preventing the formation of tumors including urothelial bladder cancer [35, 36]. Tumors with mutations in TP53 gene often exhibit accelerated progression, show limited responsiveness to anticancer treatments, such as chemotherapy, and are associated with an unfavorable prognosis [37, 38]. TP63, belonging to the p53 gene family together with p73, encodes different proteins capable of either activating p53-responsive genes or acting as a dominant-negative factor against p53 [39]. p63 shows a vital function in the typical development and upkeep of the human urothelium [40]. However, regarding bladder carcinoma, the impact of mutations in the p63 gene remains controversial for the scanty information available. While certain studies have reported a decrease in muscle-invasive tumors [41], others have shown persistent, retained expression associated with biological aggressiveness, implying a possible involvement in tumor progression [42]. However, the prospect of better understanding the role of p53 and p63 in cancer progression emerges as a potential appealing future strategy for several solid cancers [43].

Dysregulation of Notch3 has been associated with numerous cancers [44, 45], impacting tumor aggressiveness, maintenance, and resistance to chemotherapy [46–48]. In addition, studies found that NOTCH3 can have a role in orchestrating antitumoral activity by inducing the expression of PD-1 [49, 50]. According to this, mutations in NOTCH3 could be related to the high immune-checkpoint expression observed in our urothelial bladder cancer thus facilitating immune cancer escape. Notably, Notch3 plays a role in regulating p53 at a post-transcriptional level, thereby regulating Cyclin-G1 expression [51]. In more specific terms, the reduction of Cyclin-G1 in cells Notch3 knockdown leads to a substantial increase in p53 protein expression. Consequently, the accumulated p53 in Notch3-depleted cells triggers an overexpression of miR-221, resulting in enhanced p53 stability. In this context, mutations in the NOTCH3 gene may contribute to the dysregulation of p53/p63 molecules as described below. The increasing understanding of the role of the NOTCH family in cancer has heightened interest in targeting Notch molecules therapeutically. This interest extends to both single-agent approaches and multimolecular-targeted strategies. Recent investigation on Notch signaling has led to the emergence of more specific therapeutic strategies for inhibiting Notch, including for example: (i) inhibition of the nuclear transcriptional Notch coactivator (ii) neutralization with monoclonal antibodies, or receptor decoys, of the ligand–receptor interaction (iii) use small molecular GSIs to inhibit the proteolytic cleavage/activation of the receptor [52, 53]. However, at the current state of the art, there are no clinical trials specifically addressing the use of targeted therapies in NOTCH3-mutated cancers. Currently, NOTCH3 mutations serve as inclusion eligibility criteria for clinical trials evaluating the efficacy of the following anti-cancer treatments: Al101, abemaciclib, carboplatin, cisplatin, and crenigacestat [54].

The molecular characteristics of the clinical case reported here also include the identification of mutational signatures in bladder cancers: almost all detected mutations contribute to signatures 2 and 13 (Fig. 2B). These signatures are present in more than 70% of bladder cancers [55]. Such molecular signatures are commonly associated with APOBEC-mediated mutagenesis, which is linked to the activity of base excision repair and DNA replication machineries [55] opening the possibility for the use of biological therapies such as ATR inhibitors [56].

**Fig. 3:**
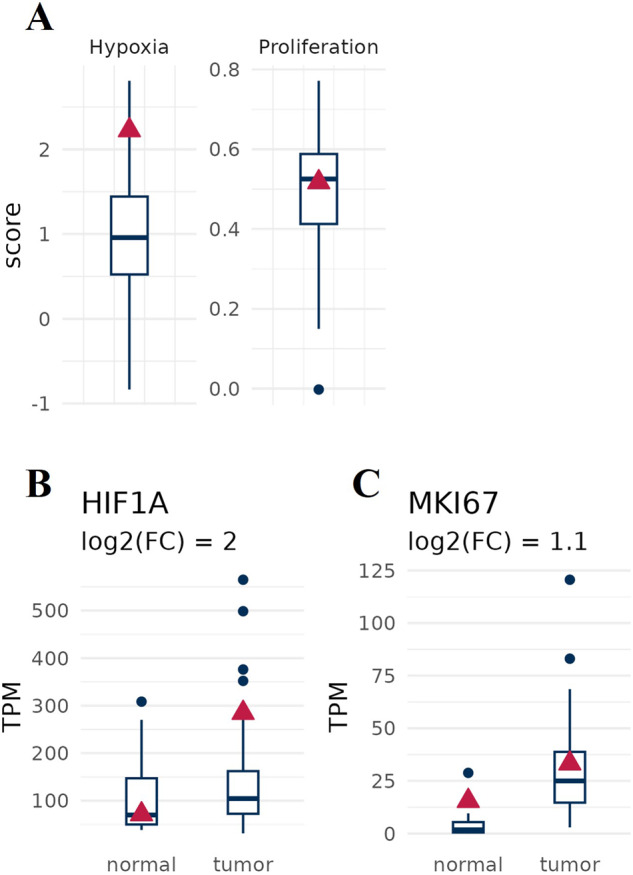
Cancer hallmark expression signatures. **A** Very high hypoxia and proliferation signature scores in a case of urothelial bladder cancer. **B**, **C** Graphs show HIF1α (**B**) and MKI67 (**C**) expression in investigated bladder tumor as compared to both normal tissue and bladder cancer background cohort. Boxplots indicate the values of bladder cancer background cohort, and the red triangle refers to our patient of interest.

The identification of very high hypoxia signature scores (Fig. 3A), as well as the up-regulation of HIF-1α and MKI67 (Fig. 3A, B), assessed by gene expression analysis, adds another layer of complexity to the case. Indeed, hypoxia in bladder cancer is linked the initiation of epithelial-mesenchymal transition, a biological phenomenon associated to cancer metastasis [57–60], suppression of apoptosis [61–64], and the advancement of cancer. Its negative impact on immunotherapy is notable, as it modifies molecular markers, immune cell movement, and angiogenesis, leading to immunosuppression via a HIF-1-dependent signature [65]. Thus, high hypoxia levels are associated with an unfavorable prognosis in bladder cancer [66]. In this context, hypoxia can influence the function of p53 in a manner that is dependent on the specific context [66–70]. In particular, hypoxia might induce inhibitory interactors of p53, including murine double minute 4 (MDM4) or MDM2, resulting in a lower p53 steady state protein level. Moreover, HIF-1α plays a role in transcriptionally activating targets that facilitate the proteasomal elimination of homeodomain-interacting protein kinase 2 (HIPK2), that normally should phosphorylate and, hence, activate the function of p53 [71]. As a result, all these concerted actions are able to significantly reduce the function of p53, that reducing programmed cell death and allowing tumor cells to grow.

Paradoxically, hypoxia condition can also result in the stabilization and activation of p53, leading to enhanced protein steady state levels which, in turn, allows the activation of secondary transcriptional target genes able to regulate DNA damage response, programmed cell death and cell cycle arrest [72]. Interestingly, the extent of p53 stabilization caused by the hypoxic microenvironment appears to be directly related to the extent hypoxia, with more pronounced effects observed under conditions of profound compared to mild hypoxia. The presence of mutations in the TP53 and NOTCH3 genes and the observed high hypoxia score, targeting p53 and/or hypoxia represent a personalized medicine option for the profiled bladder cancer. This could be especially relevant in combination with immunotherapy, given the observed high expression levels of PDL-1, PDL-2, and PD1and high TMB [73, 74].

## Conclusion

In the era of 4 P (Predictive, Personalized, Preventive and Participatory) medicine [75–78], the molecular profiling of single cancer entity represents a great opportunity for the development of patient-tailored therapies. Hence, this case report highlights the complex molecular landscape of an infiltrating urothelial bladder cancer characterized by very high TMB. In particular, multi-omics investigations revealed a peculiar molecular profile including alterations in genes and pathways related to cancer progression such as immune-checkpoint inhibitors, hypoxia, TP53, TP63 and NOTCH3. The detailed molecular characterization of this case emphasizes the potential for personalized medicine in bladder cancer treatment paving the way for targeted therapies.

## Methods

### Collection of samples

Tumour tissues collection was performed using standardized protocol aimed at preventing cold ischemia until freezing in liquid nitrogen [79–81]. Hematoxylin and Eosin (H&E) stained serial sections were used for pathological quality control (QC). Inclusion criteria for tumour samples collection: tumor content of >=30%; Necrosis <=30%; presence of invasive tumour cells. Adjacent normal tissues were also collected. Protein lysate preparation and nucleic acid extraction were performed by using 10 mg of each collected tissue [82–85]. The tissues stay frozen during the entire process.

For histological and immunohistochemical analysis, serial sections from formalin-fixed and paraffin-embedded (FFPE) blocks were used [82, 86–88]. Histological analysis was conducted by two independent pathologists on H&E-stained slides. Serial sections were used to study the expression of the main relevant prognostic and predictive biomarkers of bladder cancer by immunohistochemistry including p63, p40, p16 and GATA3 [89]. Immunohistochemical reactions were performed by using the automated Leica Bond IHC platform (Leica Biosystems, Deer Park, IL) with the following primary antibodies: mouse monoclonal anti-p63 (clone 7JUL; pre-diluted; Leica Biosystems), mouse monoclonal anti-p40 (clone BC28; pre-diluted; Leica Biosystems), mouse monoclonal anti-p16 (clone 6H12; pre-diluted; Leica Biosystems) and mouse monoclonal anti-GATA3 (clone L50-823; pre-diluted; Leica Biosystems).

### Nucleic acid extraction and quality assessment

As previously described, frozen tissue slices were used for nucleic acid extraction and quality assessment [35].

### Library preparation and NGS sequencing

Libraries for whole genome sequencing (WGS) and whole transcriptome sequencing were performed as previously described [90, 91].

### NGS data processing

To align NGS data, Grch38 genome assembly was used as reference. As concern the normal samples, the Haplotype Caller from the Genome Analysis Toolkit (GATK) was used for both identification and annotation of short genomic variations. WGS somatic variations were called using a consensus of Mutect2 [92], Strelka [93], Varscan [94] and Somatic Sniper [95]. Structural variations were called using R packages TitanCNA [96], DellyCNV and DellyCall [97], as well as Manta [98]. RNA-Seq differential expression was based on normalized readcount data (TPM: transcripts per million).

### Bioinformatical analyses

Mutational signatures were calculated using the R package MutationalPatterns [99–102]. MSI classification was done using R package MSIseq [103]. Metrices to define chromosomal instability were determined using R package CINmetrics [104] and CNHplus [105]. Aneuploidy events were analysed using ASCETS [106]. Aneuploidy event span more than 90% of the chromosome. Visualization of results was done in IGV [107]. TMB was calculated as the number of non-synonymous mutations of protein coding genes divided by exome size in Megabases.

### Supplementary information

Legend of the supplementary figure 1

Supplementary Figure 1.

## Data Availability

The data will be made available upon reasonable request.
