# Supplementary material for: Molecular profiling of a bladder cancer with very high tumour mutational burden
Source: Cell Death Discov. 2024 Apr 30;10:202. doi: 10.1038/s41420-024-01883-x (PMC11061316; doi:10.1038/s41420-024-01883-x)
Supplement: Supplementary file 1 — Legend of the supplementary figure 1 [file 41420_2024_1883_MOESM1_ESM.docx]

**Supplementary Figure 1.** RNA Seq analysis of CDKN2A (a) CDKN2B (b) and MTAP (c) in a urothelial tumor tissue compared to normal one. Boxplots indicate the values of bladder cancer background cohort, and the red triangle refers to our patient of interest.
