# Supplementary figures and images for: Molecular profiling of a bladder cancer with very high tumour mutational burden
Source: Cell Death Discov. 2024 Apr 30;10:202. doi: 10.1038/s41420-024-01883-x (PMC11061316; doi:10.1038/s41420-024-01883-x)

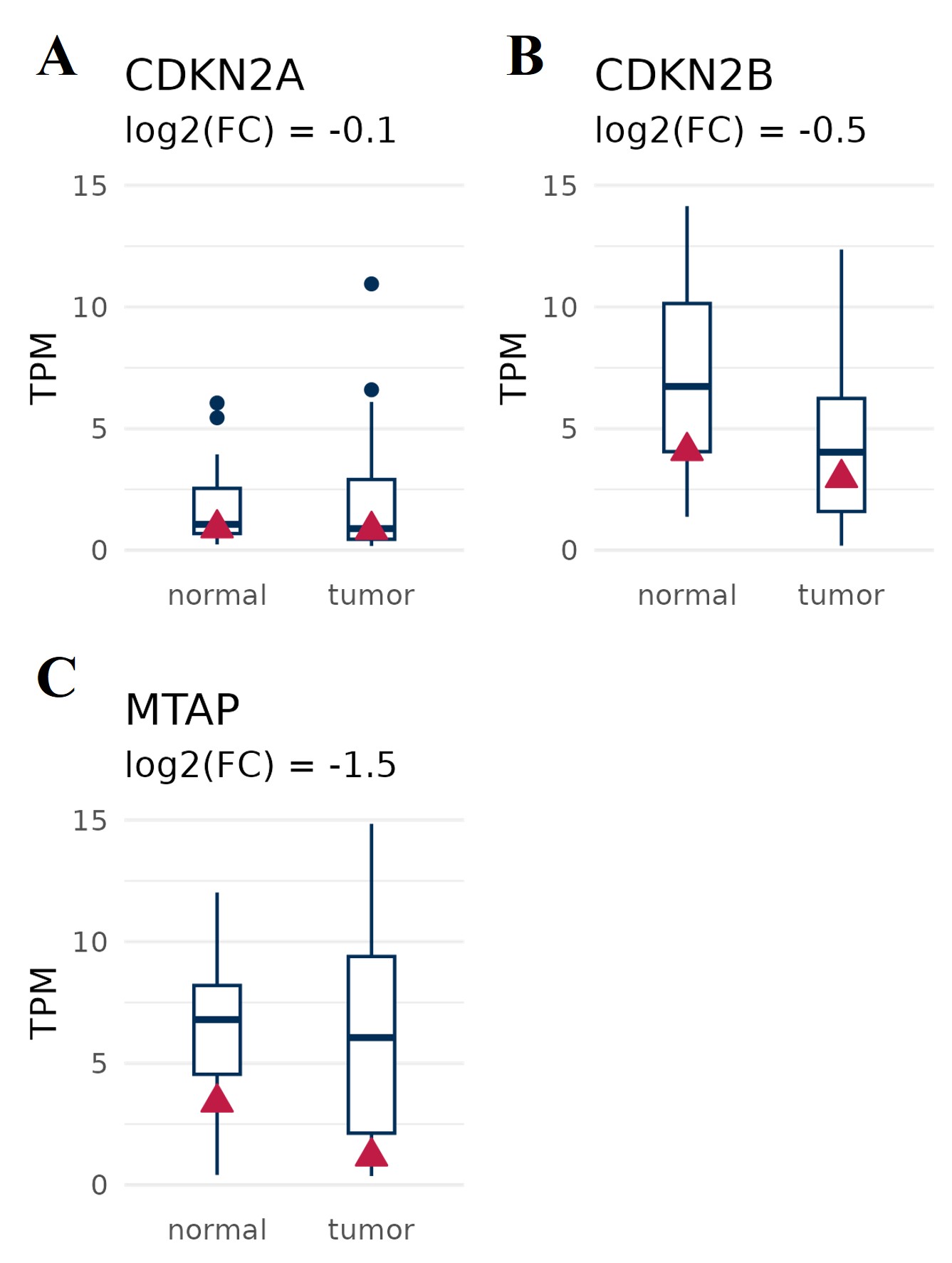

Supplement: Supplementary file 2 — Supplementary Figure 1. [file 41420_2024_1883_MOESM2_ESM.jpg]
